# Amygdalin isolated from *Semen Persicae* (*Tao Ren*) extracts induces the expression of follistatin in HepG2 and C2C12 cell lines

**DOI:** 10.1186/1749-8546-9-23

**Published:** 2014-09-16

**Authors:** Chuanbin Yang, Xuechen Li, Jianhui Rong

**Affiliations:** 1 School of Chinese Medicine, Li Ka Shing Faculty of Medicine, The University of Hong Kong, 10 Sassoon Road, Pokfulam, Hong Kong, China; 2 Department of Chemistry, The University of Hong Kong, Hong Kong, China

## Abstract

**Background:**

The Chinese medicine formulation ISF-1 (also known as *Bu-Yang-Huan-Wu-Tang*) for post-stroke rehabilitation could increase the expression of growth-regulating protein follistatin by approximately 4-fold. This study aims to identify the active compounds of ISF-1 for the induction of follistatin expression.

**Methods:**

Active compounds in ISF-1 responsible for induction of follistatin were identified by a bioactivity-guided fractionation procedure involving liquid-liquid extraction, HPLC separation and RT-PCR detection. The aqueous extracts of seven ISF-1 ingredients including S*emen Persicae* (*Tao Ren*) and the S*. Persicae*-derived fractions were assayed for the induction of follistatin mRNA expression in human hepatocarcinoma HepG2 cells by RT-PCR. The concentrations of isolated compounds were proportionally normalized to the reported IC_50_ concentration (5.8 mg/mL) of the formulation ISF-1 in HepG2. The active fractions were characterized by reverse-phase HPLC on a C18 column and identified by mass spectrometry.

**Results:**

Three ingredients of ISF-1, namely *S. Persicae* (*Tao Ren*), *Pheretima* (*Di Long*), and *Flos Carthami* (*Hong Hua*), induced the expression of follistatin mRNA. Among these, the ingredient S*. Persicae* were the most active, and amygdalin from *S. Persicae* extract was identified as a novel follistatin inducer. Amygdalin stimulated the growth of skeletal muscle cell line C2C12 cells in a concentration-dependent manner.

**Conclusions:**

Amygdalin isolated from *S. Persicae* extract in ISF-1 through a bioactivity-guided fractionation procedure induced the expression of follistatin in HepG2 and C2C12 cell lines.

## Background

Stroke causes severe disability and mortality worldwide, characterized by dramatic structural and metabolic changes in skeletal muscle that can lead to post-stroke sarcopenia [1,2]. Recombinant tissue plasminogen activator is the only FDA approved drug for the treatment of acute ischemic stroke but is limited by a narrow therapeutic window and the ineffectiveness for post-stroke recovery [3,4].

Myostatin has emerged as a molecular target for therapeutic intervention [5]. Both pharmacological inhibition and genetic targeting of myostatin were under evaluation for treating sarcopenia [6-9]. Follistatin is an autocrine glycoprotein with strong ability to antagonize different members of the TGF-β superfamily, such as activin and myostatin [10-13], regulating cell proliferation, cell differentiation, inflammatory responses, and embryogenesis [11,14-16]. Follistatin has been recently evaluated as a treatment for fibrotic diseases in animal models aa [14,17,18]. However, as an endogenous antagonist of myostatin, follistatin acts as a negative regulator of muscle growth [13]. Transfection of follistatin cDNA construct for increasing follistatin protein expression could increase muscle mass in experimental animals [6,7,19]. Therefore, pharmacological induction of follistatin expression might serve as a new strategy for the treatment of post-stroke and age-related sarcopenia.

The Chinese medicine formulation ISF-1 (also known as *Bu-Yang-Huan-Wu-Tang*), was used for post-stroke rehabilitation. The formulation consists of seven ingredients, including *Radix Astragali* (*Huang Qi*), *Radix Angelicae Sinensis* (*Dang Gui*), *Radix Paeoniae Rubra* (*Chi Shao*), *Rhizoma Chuanxiong* (*Chuan Xiong*), *Flos Carthami* (*Hong Hua*), *Semen Persicae* (*Tao Ren*), and *Pheretima* (*Di Long*) [20,21]. We previously profiled the cellular responses to formulation ISF-1 in human HepG2 cells by a genome-wide biological response fingerprinting (BioReF) approach [20], and developed a bioactivity-guided fractionation procedure involving liquid-liquid extraction, HPLC separation and RT-PCR detection to identify the active compounds involved in the induction of heme oxygenase-1 and leukotriene B4 12-hydroxydehydrogenase [22,23].

This study aims to identify the active compounds of ISF-1 for the induction of follistatin expression.

## Methods

### Cell culture and reagents

The human hepatocellular carcinoma cell line HepG2 [ATCC No. HB-8065] and mouse skeletal muscle cell line C2C12 [ATCC No. CRL-1722] were obtained from American Type Culture Collection (USA). The cells were cultured in Dulbecco’s modified Eagle’s medium supplemented with 10% (v/v) fetal bovine serum and 1% penicillin/streptomycin (Invitrogen, USA) at 37°C in a humidified atmosphere containing 5% CO_2_ and 95% air. All chemicals were obtained from Sigma-Aldrich Co. (USA), unless otherwise indicated. Dried herbal extracts, *R. Astragali* (Cat No. 1008; Batch No. A090611-01), *S. Persicae* (Cat No. 1106; Batch No. A06273-01), *Pheretima* (Cat No. 1118, Batch No. A06314-01), *F. Carthami* (Cat No. 1297, Batch No. A04579-02), *R. Sinensis* (Cat No. 1187, Batch No. A00397-03), *R. Rubra* (Cat No. 1059, Batch No. A06001-01), and *R. Chuanxiong* (Cat No. 1019; A06430-01), were purchased from Nong’s Pharmaceutical Ltd. (Hong Kong). Oligonucleotide primers specific for follistatin and glyceraldehyde 3-phosphate dehydrogenase (GAPDH) were purchased from the Centre for Genomic Sciences, The University of Hong Kong.

### Apparatus and chromatographic conditions

HPLC analysis was performed on an Alltima™ HP C18 column (250 × 4.6 mm, 5 μm, Alltech, USA) under the control of a HPLC system (Waters, USA) equipped with a Waters 996 Model photodiode array detector (DAD), a Waters 600S Model system controller, and a gradient generator. The column was run in a gradient mobile phase generated by mixing an aqueous solution (A) and acetonitrile (B). The samples were eluted with 5% B for 5 min, followed by a linear gradient up to 95% B over a period of 50 min. The flow rate was set at 1 mL/min and the column temperature was maintained at 25°C. For analysis of the fifth fraction (fraction F5) and the spiked-in amygdalin standard, the samples were eluted with aqueous solution A containing 9% acetonitrile and 9% methanol. The UV absorbance was recorded over the range of 200–400 nm.

### Assay of herbal extracts for induction of follistatin

Dried powder samples of aqueous herbal extracts (*R. Astragali*, *S. Persicae*, *Pheretima*, *F. Carthami*, *R. Sinensis*, *R. Rubra*, and *R. Chuanxiong*) were purchased from Nong’s Pharmaceutical Ltd. (Hong Kong). For herbal extract preparation, the dried powder of each herbal extract (100 mg) was dissolved in 1 mL of Milli Q water produced by Milli Q Synthesis A10 Water Purificaiton System (EMD Millipore, Germany) at 80°C for 30 min, with vortexing every 10 min. After cooling to room temperature, the insoluble materials were removed by centrifugation at 12,000 rpm on an Eppendorf 5424 microcentrifuge (Eppendorf AG, Germany) for 10 min. The supernatant was recovered and sterilized by passage through a syringe filter with a 0.22-μm (Pall, New York, USA) membrane. For assays of follistatin induction, HepG2 cells were treated for 24 h with the supernatants of the individual herbal extracts at the following concentrations: *R. Astragali*, 5.18 mg/mL; *S. Persicae*, 0.13 mg/mL; *Pheretima*, 0.13 mg/mL; *F. Carthami*, 0.13 mg/mL; *R. Sinensis*, 0.26 mg/mL; *R. Rubra*, 0.19 mg/mL; *R. Chuanxiong*, 0.13 mg/mL. The expression of follistatin was detected by RT-PCR and analyzed by 1% agarose gel electrophoresis.

### Bioactivity-guided fractionation of *Semen Persicae* extract

The dried *S. Persicae* extract (50 g) was resuspended in 250 mL of Milli Q water and heated at 80°C for 1 h. Following precipitation with 70% ethanol overnight and removal of insoluble materials by centrifugation at 4000 × *g* for 15 minutes, the supernatant (A) was collected and sequentially extracted three times with 250 mL of ethyl acetate (EA fraction) and n-butanol (n-butanol fraction). The solvents for each extraction were finally removed by a rotary evaporator (Tokyo Rikakikai, Japan) under vacuum. The dried residues from each preparation were dissolved in dimethyl sulfoxide, filtered through a 0.22-μm membrane (Pall, USA), and subjected to the follistatin induction assay in HepG2 cells.

For isolation of the active compounds, the n-butanol fraction was further separated by semi-preparative reverse-phase HPLC on an Alltima C-18 column (250 × 10.0 mm, 5 μm, Alltech, USA). The column was run in a gradient mobile phase generated by mixing an aqueous solution (A) and acetonitrile (B). The samples were eluted with 5% B for 5 min, followed by a linear gradient elution up to 95% B over a period of 50 min. The flow rate was set at 3 mL/min and the column temperature was maintained at 25°C. The UV absorbance was monitored on a HPLC UV detector (Waters, USA) over the wavelength range of 200–400 nm. Eleven fractions were collected and assayed for follistatin induction. Finally, each active compound was purified as a single peak in the HPLC profile.

### Chemical identification by mass spectrometry

The HPLC fraction containing the active compound was analyzed on a Varian MicroSorb C18 column (2 × 150 mm) (Agilent, USA) at a flow rate of 0.8 mL/min under the control of a LC-MS system (Waters, USA) equipped with a 1525 Separations Module and a Waters 2998 DAD Unit (Waters, USA). The mobile phase compositions were water with 0.3% (v/v) formic acid (A) and acetonitrile (B). The gradient was set as follows: 0–8 min, 5- 50% B; 8–10 min, 50% B; 12–15 min, 95% B. The flow rate was constant at 0.8 mL/min and the column temperature was maintained at 20°C. The injection volume was 20 μL. The eluents were analyzed on a triple quadrupole mass spectrometer 3200 QTRAP^®^ system (ABI/Sciex, USA) equipped with an ESI-Turbo V™ source operating in the positive ionization mode under the control of an Analyst v1.4.2 data system (Applied Biosystems/MDS Sciex, Canada). The mass spectrometry analyses were performed under the following conditions: N_2_ drying gas, 10 L/min; capillary voltage, 20 V; nebulizer pressure, 30 psi; ion spray voltage, 4 kV; and capillary temperature, 325°C.

### RT-PCR detection

Following treatment with herbal extracts or isolated fractions, total RNA was isolated from the cells by TRIzol reagent (Invitrogen, USA). The mRNAs were converted into cDNAs by Moloney Murine Leukemia Virus (M-MuLV) reverse transcriptase kit (Fermentas, USA). Human and mouse follistatin mRNAs were detected by PCR using specific oligonucleotide primers as follows: human follistatin mRNA (NM_006350): sense, 5'-GTTTTCTGTCCAGGCAGCTCCACA-3', antisense, 5'-GCAAGATCCGGAGTGCTTTACTTCCA-3'; mouse follistatin mRNA (NM_008046): sense, 5′-CTCTTCAAGTGGATGATTTTC-3', antisense, 5′-ACAGTAGGCATTATTGGTCTG-3′. As an internal control, GAPDH mRNA (NM_001256799) was also detected using specific oligonucleotide primers: sense, 5'-CAAGGTCATCCATGACAACTTTG-3', antisense, 5'-GTCCACCACCCTGTTGCTGTAG-3'. The PCR amplifications were performed as follows: denaturation at 94°C for 3 min; 30 cycles of amplification at 94°C for 30 s, 65°C (human follistatin), 50°C (mouse follistatin), or 55°C (GAPDH) for 30 s, and 72°C for 30 s; and extension at 72°C for 10 min. The PCR products were analyzed by electrophoresis in a 1.0% agarose gel containing Gel Red and visualized under UV light by a GelDoc imaging system (Bio-Rad, USA). The intensity of EtBr-stained gel bands was obtained by densitometric analysis using software Quantity One (Bio-Rad, USA). After normalization against housekeeping gene GAPDH, the mean values of follistatin induction relative to the untreated control were calculated based on the results of three independent experiments.

### Cell viability assay

Cell viability was evaluated by a standard colorimetric assay as previously described [24]. Briefly, C2C12 cells (0.4 × 10^4^ cells/100 μL) were seeded in a 96-well microplate and treated with amygdalin at concentrations of 0–25 μM for 24 or 48 h. At the end of the treatment, the cell monolayers were incubated with 3-[4,5-dimethylthiazol-2-yl]-2,5-diphenyltetrazolium bromide (MTT) solution (0.5 mg/mL) in phosphate-buffered saline for 4 h. The formation of purple formazan was measured in relation to the absorbance at 570 nm by a microplate reader (Bio-Rad, USA). The cell viability was presented as a percentage relative to that of vehicle-treated control cells.

### Statistical analysis

The results were presented as means ± SD from three independent experiments. Statistical analysis was performed by two-tailed unpaired Student’s *t*-test with SPSS 13.0 software (SPSS, Chicago, USA). A *P* value < 0.05 was considered as statistically significant.

## Results

### Screening of herbal extracts for induction of follistatin expression

**Figure 1:**
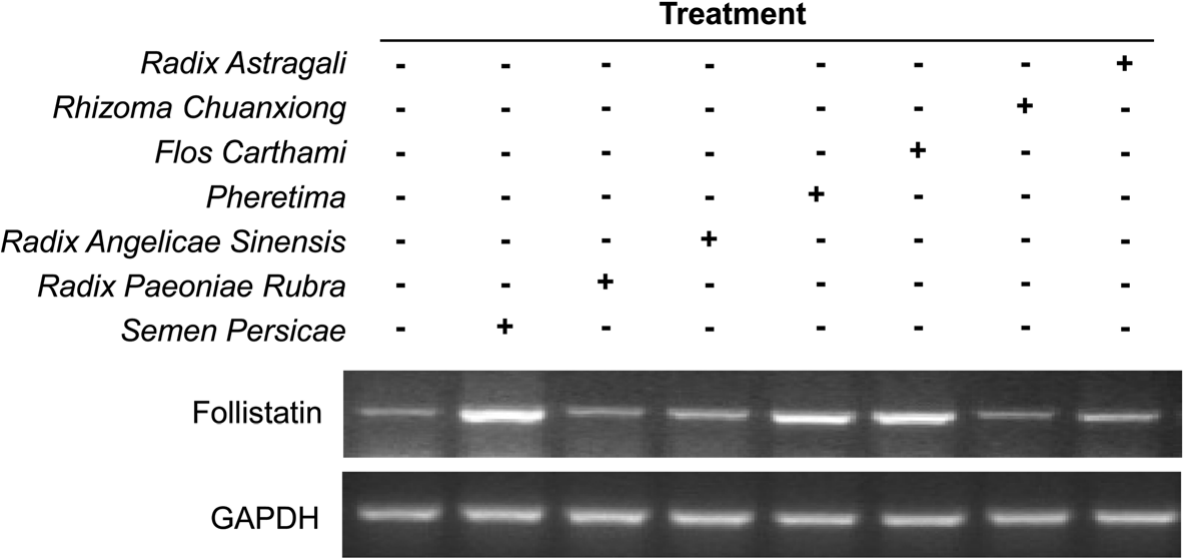
**Identification of the active ingredients for induction of follistatin expression.** HepG2 cells were treated with different herbal extracts, *R. Astragali*, *R. Chuanxiong*, *F. Carthami*, *Pheretima*, *R. Sinensis*, *R. Rubra*, and *S. Persicae*, for 24 h. Subsequently, the expression of follistatin mRNA was detected by RT-PCR and 1 % agarose gel electrophoresis of the PCR products. +, treatment with selected herbal extract; -, no treatment with selected herbal extract.

We treated HepG2 cells with aqueous extracts of the individual herbs for 24 h. Follistatin induction was subsequently detected by RT-PCR using specific primers. Three herbal extracts (*S. Persica*e, *Pheretima*, and *F. Carthami*) induced the expression of follistatin mRNA, while the expression of the housekeeping gene GAPDH was not affected (Figure 1). The *S. Persicae* extract showed the strongest activity for increasing follistatin mRNA expression among the three extracts. Thus, the *S. Persicae* extract was selected for further characterization by bioactivity-guided fractionation.

### Bioactivity-guided fractionation of *S. Persicae* extract for follistatin induction

**Figure 2:**
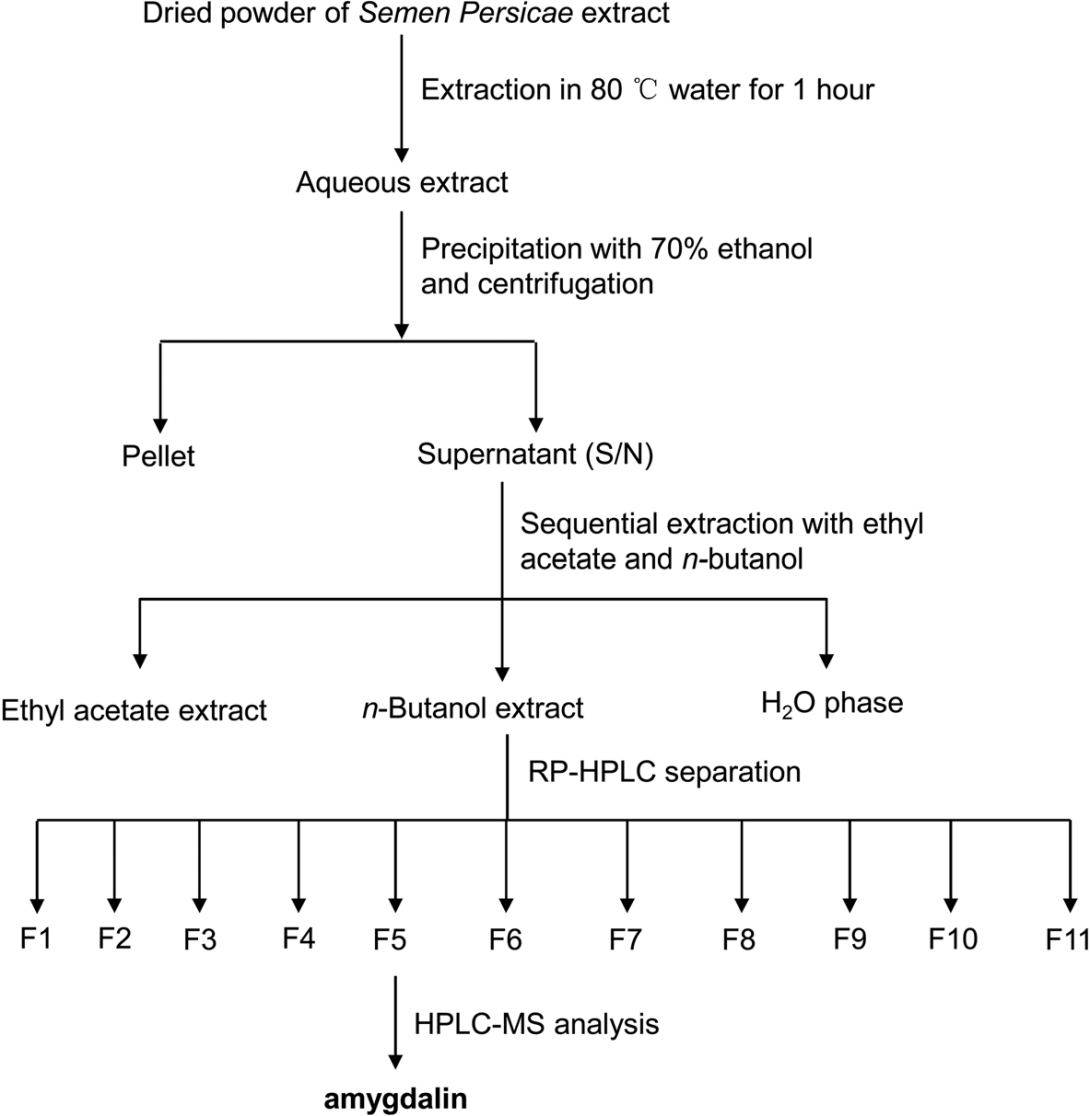
**Schematic illustration of the bioactivity-guided fractionation procedure for identification of the active compound from *S. Persicae *extract.** The n-butanol extract was separated by semi-preparative RP-HPLC on a C18 column. All of the fractions were analyzed by semi-quantitative RT-PCR for follistatin mRNA induction in HepG2 cells.

**Figure 3:**
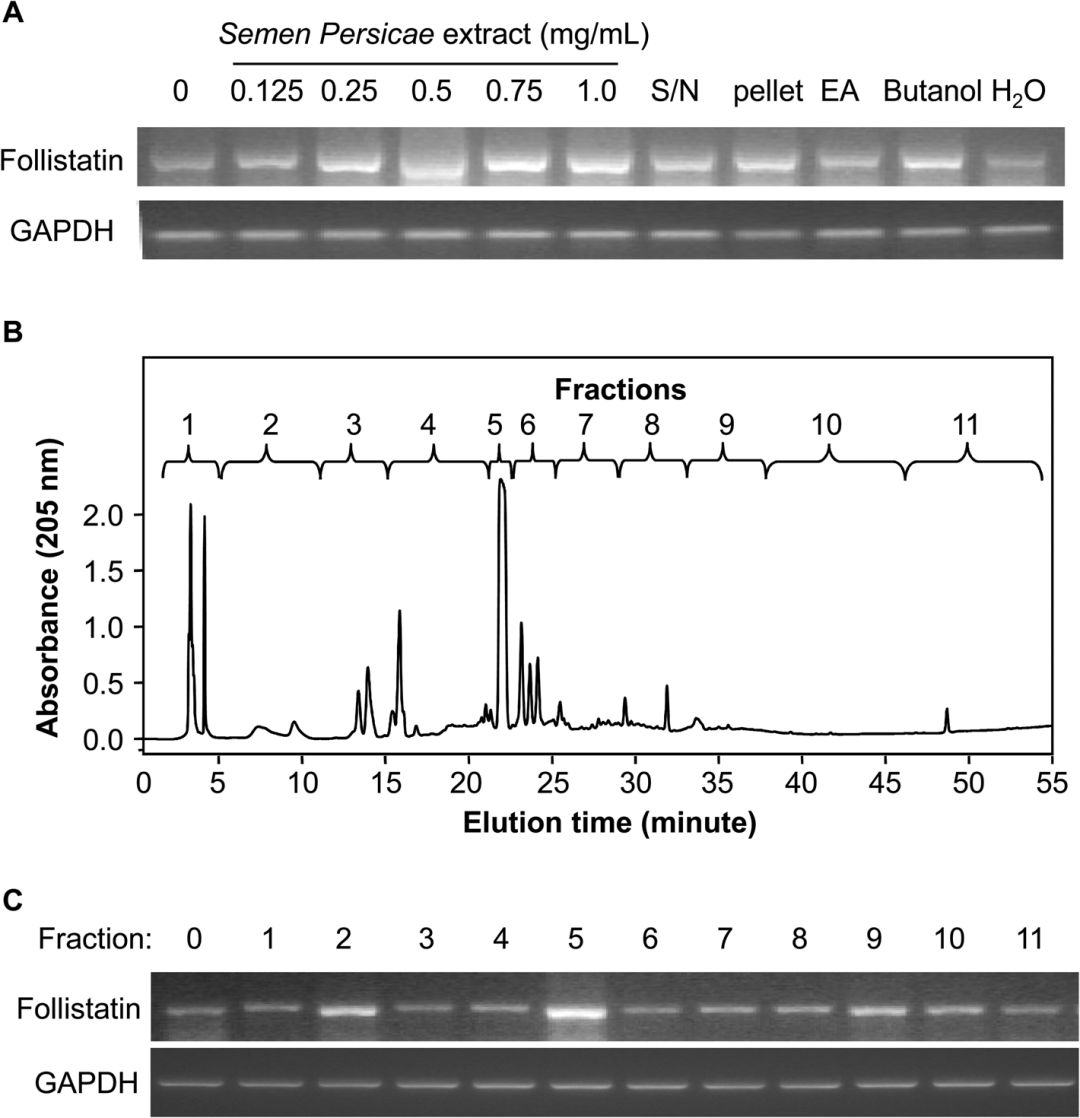
**RT-PCR assay for induction of follistatin expression. (A)** Assays of *S. Persicae* extract and liquid-liquid extraction products. HepG2 cells were treated with *S. Persicae* extract at the indicated concentrations and *S. Persicae*-derived fractions for 24 h, and the expression of follistatin mRNA was detected by RT-PCR. S/N, supernatant; EA, ethyl acetate. **(B)** HPLC separation of the n-butanol extract. The n-butanol extract was separated by semi-preparative RP-HPLC on a C18 column into 11 fractions. **(C)** Assays of HPLC fractions. The HPLC fractions were assayed for induction of follistatin mRNA expression.

We developed a simple bioactivity-guided fractionation procedure to isolate the active compounds from *S. Persicae* extract (Figure 2). The dried aqueous herbal extract purchased from Nong’s Pharmaceutical Ltd. and was verified to have activity in follistatin induction. Briefly, HepG2 cells were treated with *S. Persicae* extract at contractions of 0, 0.125, 0.25, 0.5, 0.75, and 1.0 mg/mL. Subsequent RT-PCR analyses of follistatin mRNA confirmed *S. Persicae* extract induced the expression of follistatin mRNA in a concentration-dependent manner (Figure 3A). For bioactivity-guided fractionation, the dried *S. Persicae* extract was re-extracted and then precipitated with ethanol. The resulting mixture was separated by centrifugation into two parts, a supernatant (S/N) and a pellet. The supernatant was sequentially extracted with ethyl acetate (EA) and n-butanol, giving rise to another three fractions, EA extract, n-butanol extract, and H_2_O phase. All of the fractions were evaluated for induction of follistatin mRNA. The n-butanol extract effectively induced the expression of follistatin mRNA (Figure 3A).

### Amygdalin in the n-butanol extract of *S. Persicae* is a novel inducer of follistatin

**Figure 4:**
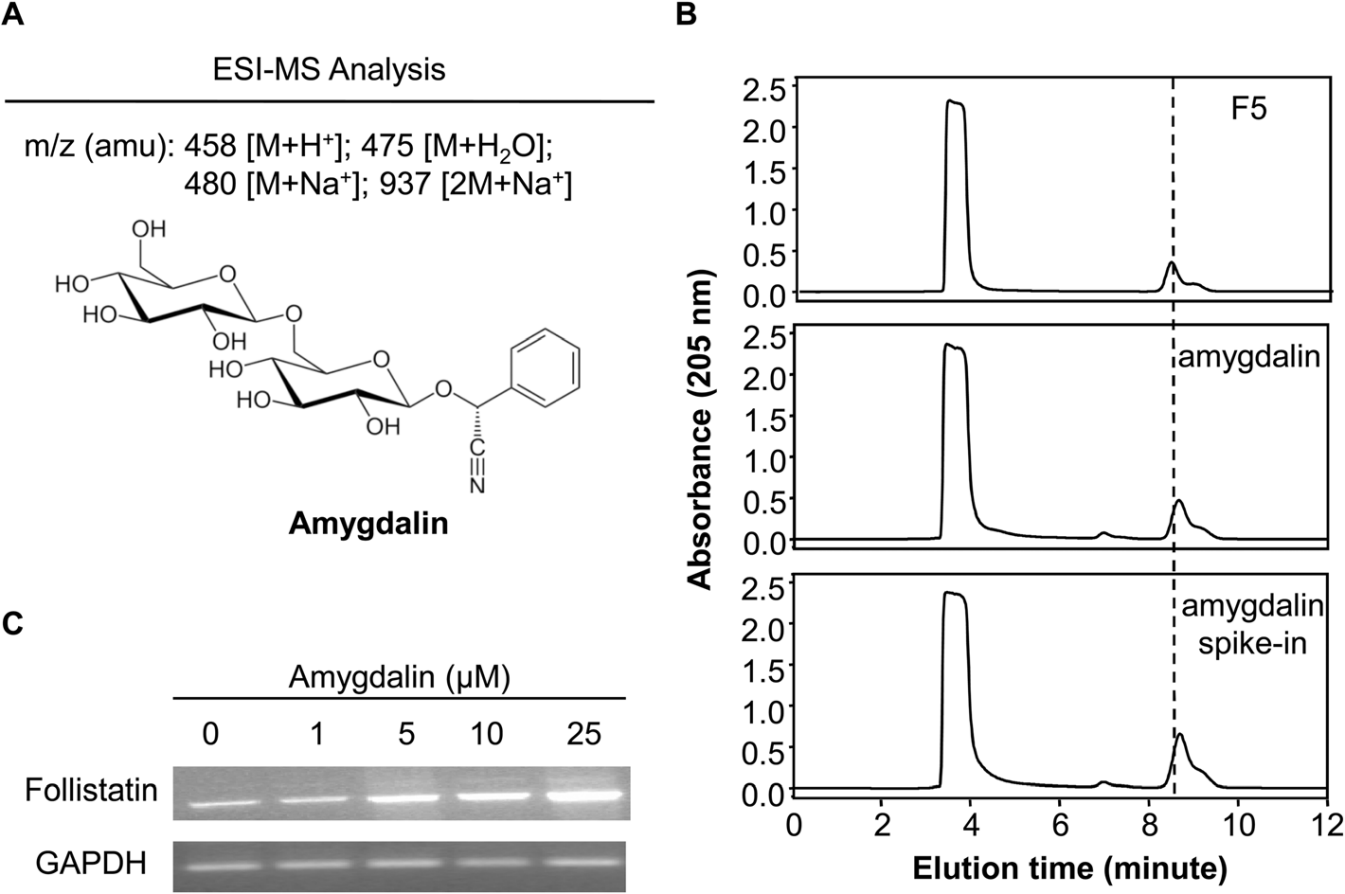
**Chemical characterization of fraction F5. (A)** LC-MS analysis of fraction F5. Positive ESI-MS *m/z* signals are shown. The chemical structure of amygdalin was generated by ACD/Chem Sketch software (http://www.acdlabs.com/resources/freeware/chemsketch/). **(B)** HPLC verification of amygdalin as the active compound. The fraction F5, amygdalin standard, and F5-amygdalin mixture were sequentially analyzed by reverse-phase HPLC on a C18 column under the same conditions. **(C)** RT-PCR verification of commercial amygdalin for induction of follistatin mRNA. HepG2 cells were treated with commercial amygdalin at the indicated concentrations for 24 h. The expression of follistatin mRNA was detected by RT-PCR.

We separated the n-butanol extraction into 11 fractions by HPLC on a C18 column to isolate the active compound from the n-butanol extract of *S. Persicae* (Figure 3B). All of the fractions were assessed for induction of follistatin mRNA. Fraction F5 showed the strongest activity in inducing follistatin mRNA expression, while fractions F2 and F9 also showed some activity (Figure 3C). Fraction F5 was further analyzed by LC-MS. The positive ESI-MS *m/z* spectrum of fraction F5 contained four important signals at 458 [M + H^+^], 475 [M], 480 [M + Na^+^], and 937 [2 M + Na^+^] (Figure 4A), suggesting the presence of amygdalin in this fraction [25].

We compared the retention times of fraction F5 and an amygdalin standard in the HPLC profile to verify the effect of amygdalin on follistatin mRNA expression. Fraction F5 was confirmed to elute with the same pattern as the commercial amygdalin standard. We observed two closely eluted peaks for both fraction F5 and the amygdalin standard (Figure 4B). Next, we clarified whether the commercial amygdalin standard could induce follistatin mRNA. As expected, the commercial amygdalin standard effectively induced follistatin mRNA expression in a concentration-dependent manner (Figure 4C). LC-MS analyses of fractions F2 and F9 could not identify any chemical compounds reported by others [26,27].

### Effects of amygdalin on the expression of follistatin and proliferation of skeletal muscle C2C12 cells

**Figure 5:**
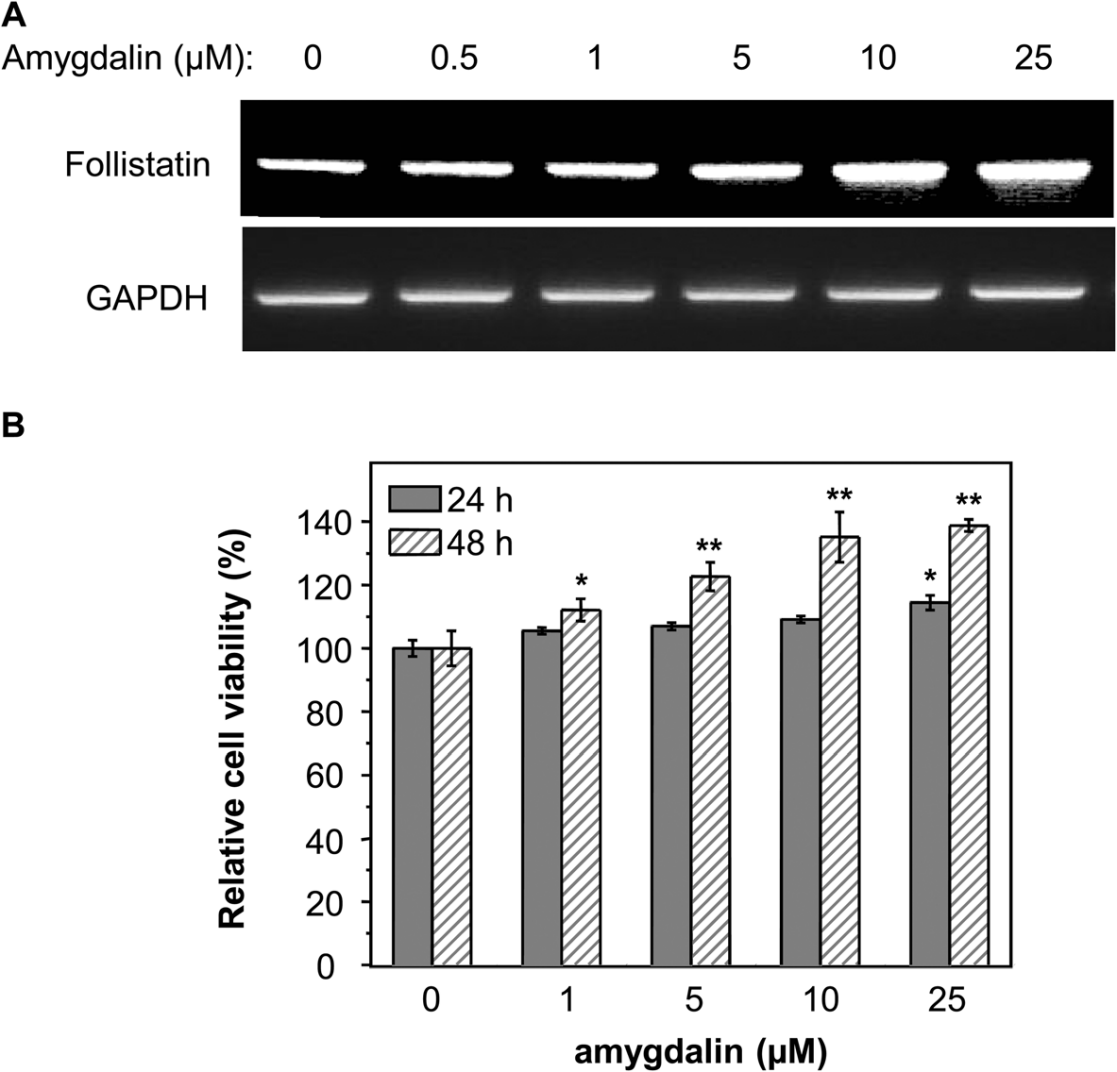
**Effect of amygdalin on follistatin expression and cell proliferation in skeletal muscle C2C12 cells. (A)** Induction of follistatin mRNA by amygdalin. Mouse skeletal muscle C2C12 cells were treated with amygdalin at the indicated concentrations for 24 h. The levels of follistatin mRNA were determined by RT-PCR. **(B)** Stimulation of skeletal muscle cell growth by amygdalin. C2C12 cells were treated with amygdalin at the indicated concentrations for 24 or 48 h. The viable cells were measured by a standard MTT assay. The values represent means ± SD from three independent experiments. *P < 0.05, **P < 0.01, amygdalin treatment *vs.* control.

We treated the cells of mouse skeletal muscle cell line C2C12 with amygdalin at various concentrations (0–25 μM) to characterize whether amygdalin could induce follistatin mRNA expression in skeletal muscle cells. The expression of follistatin mRNA was detected by RT-PCR using specific primers. Amygdalin effectively induced the expression of follistatin mRNA in a concentration-dependent manner (Figure 5A). We further investigated whether amygdalin could accelerate the growth of skeletal muscle cells. We treated C2C12 cells with amygdalin at the same concentrations (0–25 μM) for 24 or 48 h. The viable cells were determined by a standard MTT assay. Amygdalin was confirmed to stimulate the growth of skeletal muscle cell line C2C12 cells in a concentration- and time-dependent manner (Figure 5B).

## Discussion

The genome-wide BioReF approach not only helps in the selection of candidate genes for complex herbal medicines in a bias-free manner, but also enables rapid identification of the corresponding active compounds [20,22,23]. Subsequent bioactivity-guided fractionation could be performed on a relatively small scale to identify the active compounds that were actually responsible for the specific bioactivity of the parent herbal extracts. In the present study, we investigated the active compounds for the induction of follistatin, from a previous BioReF profile [20]. We developed a bioactivity-guided fractionation procedure to identify the active compound from formulation ISF-1 involved in the induction of follistatin expression. We identified three herbs, *S. Persicae*, *Pheretima*, and *F. Carthami*, as the active ingredients in HepG2 cells. Amygdalin was identified from the *S. Persicae* extract as an inducer of follistatin. Amygdalin existed in two forms, D-amygdalin and neoamygdalin [28,29]. The two closely eluted peaks in the HPLC profiles of fraction F5 and the amygdalin standard were observed, which might reflect the two forms of amygdalin. However, we cannot rule out the possibility that similar or different active compounds might be present in other active fractions (*e.g.*, fractions F2 and F9).

*S. Persicae* was an anticoagulant, antiphlogistic, and anodyne in Chinese medicine [30,31]. However, little was known about the role of *S. Persicae* in the treatment of post-stroke disorders. Several myostatin inhibitors, including myostatin propeptide, monoclonal antibodies, follistatin, and follistatin-like proteins, have been evaluated for the treatment of muscle-wasting disorders [7,13,32-34]. While these studies used recombinant follistatin as myostatin inhibitor, the present study demonstrated that amygdalin in *S. Persicae* could induce the expression of follistatin mRNA.

Two recent studies applied *S. Persicae* or *S. Persicae*-containing formulations (*e.g.*, *Fu Zheng Hua Yu* recipe) to the treatment of liver fibrosis [35,36]. Amygdalin in *S. Persicae* might be helpful in the treatment of fibrosis, according to animal experiments [37]. However, follistatin has recently emerged as an important therapeutic target in the treatment of fibrotic diseases for its potent ability to bind and neutralize activin [14,17,38].

## Conclusions

Amygdalin isolated from *S. Persicae* extract in ISF-1 through a bioactivity-guided fractionation procedure induced follistatin expression in HepG2 and C2C12 cell lines.

## Abbreviations

BioReF: Biological response fingerprinting; EA: Ethyl acetate; ESI-MS: Electrospray ionization-mass spectrometry; EtBr: Ethidium bromide; FDA: U.S. Food and Drug Administration; GAPDH: Glyceraldehyde 3-phosphate dehydrogenase; HPLC: High performance liquid chromatography; ISF-1: Ischemic stroke formulation-1; LC-MS: Liquid chromatography–mass spectrometry; mRNA: Messenger RNA; RP-HPLC: Reverse phase high performance liquid chromatography; RT-PCR: Reverse transcription-polymerase chain reaction; TGF-β: Transforming growth factor beta; UV: Ultraviolet.

## Competing interests

The authors declare that they have no competing interests.

## Authors’ contributions

JR and XL conceived and designed the study. CY performed the experiments and the statistical analysis. JR and CY wrote the manuscript. All authors read and approved the final manuscript.
